# Supplementary figures and images for: Transcription arrest induces formation of RNA granules in mitochondria
Source: Life Sci Alliance. 2025 Jun 16;8(9):e202403082. doi: 10.26508/lsa.202403082 (PMC12171109; doi:10.26508/lsa.202403082)

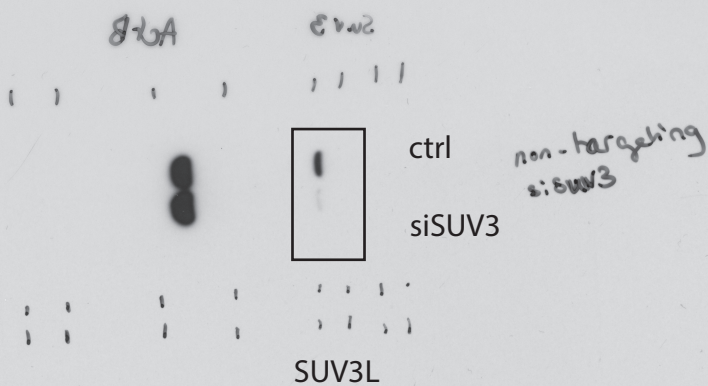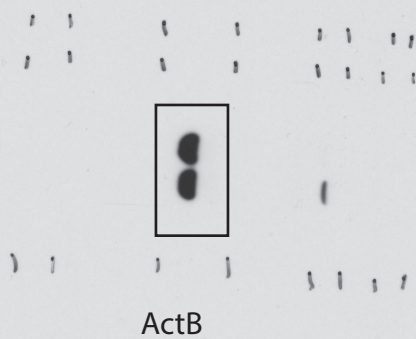

Supplement: Supplementary file 2 [file LSA-2024-03082_SdataFS5.pdf]
